# Establishing influenza epidemic thresholds and intensity levels in Türkiye using the moving epidemic method and assessing concordance with WHO transmission zones: A sentinel surveillance analysis of ten seasons, 2013–2025

**DOI:** 10.1017/S095026882610209X

**Published:** 2026-10-15

**Authors:** Eray Ontas

**Affiliations:** 1 Department of Public Health, Division of Epidemiology, Ankara University School of Medicine, Ankara, Türkiye; 2 Department of Health Informatics, Graduate School of Informatics, Middle East Technical University, Ankara, Türkiye

**Keywords:** epidemic threshold, influenza, intensity levels, moving epidemic method, surveillance, Türkiye, WHO transmission zones

## Abstract

The Moving Epidemic Method (MEM) is the ECDC/WHO-Europe-endorsed approach for establishing influenza epidemic thresholds and intensity levels, yet no positivity-based MEM analysis exists for Türkiye or any country in the WHO Western Asia transmission zone. Weekly influenza positivity rates from FluNet sentinel surveillance were analysed for ten of twelve seasons (2013/2014–2024/2025, excluding two COVID-19 pandemic seasons) using the mem R package with cross-validated optimized slope parameter, and Türkiye’s average epidemic curve and thresholds were compared with the Western Asia (excluding Türkiye) and South West Europe zones using Lin’s concordance correlation coefficient (CCC), root mean square deviation (RMSD) and complementary correlation metrics. The epidemic threshold was 10.59% positivity, with intensity thresholds at 42.56% (medium), 60.92% (high), and 71.38% (very high); cross-validated sensitivity was 0.77, specificity 0.88, and Matthews correlation coefficient 0.65. Türkiye’s average curve showed higher concordance with Western Asia (CCC=0.890; RMSD=4.22 percentage points) than South West Europe (CCC=0.672; RMSD=12.48) despite similar Pearson correlations (0.922 vs. 0.939), and the Western Asia epidemic threshold (11.50%) closely matched Türkiye’s. These first operational MEM thresholds for Türkiye and the first positivity-based thresholds for the Western Asia zone confirm alignment with the current WHO transmission zone classification and support integration into routine surveillance.

## Key Results

- First MEM-derived virological epidemic and intensity thresholds for Türkiye and Western Asia zone
- Epidemic threshold set at 10.59% positivity with medium, high, and very high levels
- Cross-validated sensitivity of 0.77, specificity of 0.88, and Matthews correlation coefficient of 0.65
- Türkiye’s epidemic curve shows higher concordance with Western Asia than South West Europe
- Concordance metrics are essential for comparing epidemic curves across transmission zones

## Introduction

Seasonal influenza remains a leading cause of acute respiratory morbidity and mortality worldwide, with an estimated 290000–650000 respiratory deaths annually and substantial economic burden through healthcare utilization and lost productivity [1]. Temperate Northern Hemisphere countries, including Türkiye, experience predictable winter epidemics typically peaking between December and March, yet the precise timing, duration, and intensity of each season vary considerably, complicating public health preparedness and response [2,3].

Effective influenza surveillance requires not only the detection of circulating viruses but also the capacity to classify each season’s severity in real time, enabling proportionate public health action. Standardized epidemic thresholds and intensity levels provide the quantitative framework for such classification, allowing surveillance authorities to distinguish baseline circulation from epidemic activity and to grade the latter into operationally meaningful severity tiers [4]. Without country-specific thresholds, surveillance remains descriptive rather than actionable.

The Moving Epidemic Method (MEM), developed by Vega et al. [5], has emerged as the standard approach for establishing influenza epidemic thresholds and intensity levels across the World Health Organization (WHO) European Region. MEM derives epidemic and intensity thresholds from historical surveillance data; the mechanics of the algorithm are described in section “The moving epidemic method” [5,6]. Following successful pilot implementation in 2010/2011, MEM was adopted by the European Centre for Disease Prevention and Control (ECDC) and the WHO Regional Office for Europe for routine influenza surveillance, and has since been applied across more than 28 European countries [6,7].

Beyond Europe, MEM has been applied in diverse settings including Australia within the WHO Pandemic Influenza Severity Assessment (PISA) framework [8], China for age-stratified threshold estimation [9], and several countries in the Eastern Mediterranean and North African region, including Egypt [10], Morocco [11], Tunisia [12], and Iraq [13]. Country-specific applications have demonstrated that epidemic thresholds vary substantially depending on the surveillance indicator used, the characteristics of the national sentinel system, and the epidemiological context, underscoring the necessity of locally calibrated thresholds rather than reliance on regional or global defaults [6,14].

Türkiye maintains an active influenza sentinel surveillance system through the Ministry of Health’s General Directorate of Public Health, contributing virological data to the WHO Global Influenza Surveillance and Response System (GISRS) via the FluNet platform since 2004. Prior epidemiological studies have characterized circulating influenza subtypes and seasonal patterns in Türkiye [15–18], and Türkiye’s sentinel system has contributed to international collaborative investigations [19]. However, no published study has applied the MEM to Turkish national sentinel surveillance data to establish epidemic thresholds and intensity levels.

This gap is particularly significant given Türkiye’s geographical position bridging Europe and Western Asia. According to the WHO influenza transmission zone classification (version dated 24 September 2025), Türkiye is assigned to the Western Asia zone alongside 17 other countries and territories: Armenia, Azerbaijan, Bahrain, Cyprus, Georgia, Iraq, Israel, Jordan, Kuwait, Lebanon, the occupied Palestinian territory including east Jerusalem, Oman, Qatar, Saudi Arabia, the Syrian Arab Republic, the United Arab Emirates, and Yemen [20]. These zones, defined as geographical groups of countries with similar influenza transmission patterns, are used to inform global surveillance reporting and vaccine strain selection timing. Within the Western Asia transmission zone, MEM thresholds have to date been published for one country only. The original multi-country MEM analysis of Vega et al. [5] analysed 20 datasets from 19 countries and regions, among them 12 seasons of sentinel influenza-like illness (ILI) data from Israel, for which a pre-epidemic threshold of 42 and a post-epidemic threshold of 47 cases per 100000 population were reported. Those thresholds were derived from ILI consultation rates rather than virological positivity, using historical data up to the 2008/2009 season and intensity levels (50%, 90%, 95%) that differ from those subsequently harmonized across the WHO European Region. No positivity-based MEM thresholds have been published for any country in the zone, and none at all for Türkiye. Data-driven analyses of influenza spatiotemporal dynamics in the WHO European Region have suggested that Türkiye clusters outside the main European transmission patterns, supporting its Western Asian classification [21,22].

The COVID-19 pandemic profoundly disrupted global influenza circulation during 2020–2022, as public health measures like masking, social distancing, and travel restrictions led to an unprecedented decline in influenza positivity rates across most countries [23,24]. The subsequent post-pandemic period has been characterized by altered seasonality and potential “immunity debt” effects [25,26]. These disruptions pose methodological challenges for MEM-based surveillance, as pandemic seasons with suppressed or absent influenza activity do not reflect typical epidemic dynamics and their inclusion can distort baseline and threshold calculations [27].

Türkiye-specific circumstances also bear on this period. In Türkiye, sentinel surveillance continued during 2020–2022, although national laboratories primarily shifted their focus towards SARS-CoV-2 testing and genomic surveillance [28]. Separately, ECDC and the WHO Regional Office for Europe now recommend that European countries maintain integrated, year-round respiratory virus surveillance rather than season-limited influenza surveillance, stating that year-round data collection and reporting should be maintained in order to identify upsurges and outbreaks during the summer months [27]. Following the pandemic, Türkiye adopted this recommendation by expanding its framework into a unified Respiratory Virus Surveillance programme. The implications of this transition for the study period are detailed in section “Data preparation and exclusions” and the Limitations.

The objectives of this study were: (1) to establish epidemic and intensity thresholds for influenza in Türkiye using the MEM applied to 10 of 12 seasons (2013/2014–2024/2025, excluding two COVID-19 seasons) of sentinel surveillance data from FluNet; (2) to evaluate the model’s classification performance through cross-validated goodness-of-fit assessment; and (3) to compare Türkiye’s MEM-derived average epidemic curve and thresholds with those of the Western Asia (excluding Türkiye) and South West Europe influenza transmission zones, thereby empirically assessing the concordance of Türkiye’s influenza dynamics with its designated WHO transmission zone.

## Materials and methods

### Data source and study period

We used sentinel influenza surveillance data from Türkiye, obtained from the WHO GISRS through the FluNet database [29]. The study period spanned 12 consecutive influenza seasons from 2013/2014 through 2024/2025, of which 10 were analysed following exclusion of the two COVID-19 pandemic seasons (2020/2021 and 2021/2022; section “Data preparation and exclusions”). Seasons were defined according to the Northern Hemisphere convention (epidemiological week 40 to week 20; 33 weeks per season).

Türkiye’s influenza sentinel surveillance was initiated in 2004 under the Communicable Disease Notification System Directive and has been operational continuously since the 2005/2006 season as the Sentinel Influenza-Like Illness (ILI) Surveillance programme [30]. The system currently comprises 250 family physicians distributed across 23 of Türkiye’s 81 provinces (20 physicians each in Istanbul and Ankara; 10 per province elsewhere). Since December 2015, Severe Acute Respiratory Infection (SARI) surveillance has been conducted in parallel to monitor hospitalized cases and associated risk factors. Since 2017, both ILI and SARI data have been collected through the web-based Public Health Management System (HSYS). Following the COVID-19 pandemic, the surveillance framework was expanded to encompass 20 respiratory viruses under the unified “Respiratory Virus Surveillance” programme, conducted through both sentinel and non-sentinel components. Laboratory diagnosis is performed at nine public health laboratories using RT-PCR as the primary method. Surveillance data are reported weekly to the ECDC European Surveillance System (TESSy) and to WHO FluNet by the Department of Communicable Diseases and Early Warning within the General Directorate of Public Health [30]. Publicly accessible sentinel positivity data on FluNet are available from the 2013/2014 season onward, which defined the starting point for the present analysis.

### Data preparation and exclusions

The primary indicator was the weekly influenza positivity rate (percentage of sentinel specimens testing positive). This indicator adjusts for variation in testing volume across weeks and seasons, providing a comparable measure of circulation intensity [14,31]. Two complete seasons (2020/2021 and 2021/2022) were excluded. In both, reported positivity ran at zero or far below the levels expected from preceding seasons – for Türkiye the 2020/2021 seasonal mean was 0.08%, with only one week recording any detection, and the comparator zones were similarly flat. These values coincide with the reprioritization of national surveillance and laboratory capacity towards SARS-CoV-2 during the pandemic and are better regarded as pandemic-affected data – reflecting reduced, interrupted or unreported sentinel testing – than as evidence of genuinely near-absent influenza circulation; they are more consistent with missing or unreliable surveillance data in FluNet than with true near-zero detection. In 2021/2022 the compared entities additionally followed markedly divergent epidemic timetables, Türkiye and Western Asia peaking in week 52 and South West Europe in week 13, a separation far larger than in any analysed season. Seasons in which the compared entities diverge to this degree cannot be meaningfully aligned by the centring algorithm on which MEM depends, and their inclusion would violate the assumption underlying the comparative design. Both seasons are nonetheless retained in Supplementary Tables S1–S3 so that readers may inspect them directly. The 2019/2020 season was truncated after epidemiological week 5, as the WHO public health emergency declaration on 30 January 2020 and the subsequent pandemic declaration on 11 March 2020 compromised sentinel data representativeness from that point onward. Missing values were handled by MEM’s internal loess imputation; week 53 data were shifted forward per MEM guidance [7].

The analysis used ILI-based sentinel data exclusively. SARI data were not included, and the two indicators were not combined at any stage. The same ILI-only filter was applied to Türkiye and to every comparator country, so that the pooled zone series are constructed on the same basis. Weeks falling outside the week 40 to week 20 window were excluded. This window was retained despite Türkiye’s transition to year-round surveillance for three reasons: MEM requires a defined surveillance period containing a single epidemic wave per season, and this is the window under which the method was developed and validated; a single consistent window across all 12 seasons is necessary for the historical comparability on which threshold estimation depends, and 8 of the 10 analysed seasons predate the transition; and the same window had to be applied to the comparator zones for the concordance analysis to be meaningful.

### The moving epidemic method

MEM operates through a three-step process [5–7]. First, the epidemic period of each season is identified using the maximum accumulated proportions (MAP) curve algorithm: the MAP curve describes the proportion of the seasonal total accumulated by successive weeks, and the epidemic boundary is placed where the slope of that curve falls below a specified value (the slope parameter). Second, an epidemic threshold is calculated from pre-epidemic values pooled across all eligible historical seasons included in the model – not from any single season. The number of pre-epidemic values taken per season is selected automatically by MEM (mem argument i.n.max = −1) according to the number of seasons supplied; for the ten-season models used here it returned three values per season, giving 30 pooled pre-epidemic observations from which the threshold is computed. Third, intensity thresholds are derived from the highest epidemic values, pooled in the same way (three peak values per season, giving 30 pooled epidemic observations), and are evaluated at three confidence levels to classify epidemic weeks into five operational levels: baseline, low, medium, high, and very high.

The method is implemented in the freely available mem package for *R* [32], and was run here through its graphical web application, memapp [7, 32]; the application, its installation and use, and its technical manual are described in Supplementary Note S1. Together with the weekly positivity series in Supplementary Tables S1–S3 and the publicly available WHO FluNet source data, this permits full reproduction of the analysis.

### MEM parameterization

The complete specification, giving for each parameter its formal mem argument name, its value and whether that value is the package default, is provided in Supplementary Table S4. Of the parameters governing the model, one was chosen empirically by the optimization algorithm; all others retained the mem package default, so the results do not depend on discretionary modelling choices.

Epidemic timing used the fixed criterium method (i.method = 2, the package default), which locates the epidemic boundary at the point where the slope of the MAP curve falls below the slope parameter. The slope parameter (i.param) was the single parameter optimized: it was selected by maximizing the Matthews correlation coefficient (MCC) across a cross-validated grid from 1.0 to 5.0 in steps of 0.1 (i.detection.values = seq(1, 5, 0.1), the package default range), independently for each entity. The optimization returned 1.8 for Türkiye and for Western Asia, and 2.3 for South West Europe. These values are lower than the package default of 2.8 and below the range of 2.5–3.0 described as typical in the package documentation. Because a lower slope parameter requires a flatter MAP curve before the epidemic is declared to have ended, it extends the identified epidemic period; this is consistent with the comparatively long average epidemic duration and high epidemic percentage obtained for Türkiye (Table 1). Seasons were centred using MEM automatic centring (i.centering = −1).

**Table 1.** Comparative MEM outputs, model performance, and concordance metrics: Türkiye, Western Asia (excluding Türkiye), and South West Europe influenza transmission zones Table 1. long description.

Parameter	Türkiye	Western Asia^a^	South West Europe
*MEM epidemic estimators*			
Seasons in model	10	10	10
Optimized slope parameter	1.8	1.8	2.3
Average epidemic start week	W49 (CI: 48–52)	W42 (CI: 40–1)	W51 (CI: 47–52)
Average epidemic duration (weeks)	20.5 (CI: 14.6–22.3)	24.5 (CI: 16.7–27.7)	17.0 (CI: 15.0–19.0)
Epidemic percentage (%)	94.12	90.95	89.11
*Epidemic and intensity thresholds*			
Epidemic threshold (%)	10.59	11.50	20.43
Post-epidemic threshold (%)	22.04	14.09	18.61
Medium intensity (%)	42.56	33.12	50.96
High intensity (%)	60.92	52.99	70.04
Very high intensity (%)	71.38	65.22	80.61
*Average curve characteristics*			
Peak positivity (%)	29.45	27.75	46.89
Peak week	W1	W3	W5
Avg curve: first week above threshold	W49	W48	W51
Avg curve: last week above threshold	W16	W14	W14
Avg curve: weeks above threshold	20	19	16
Mean of MEM average curve, W40–W20 (%)	13.66	15.33	22.32
*Model performance*			
Sensitivity	0.77	0.88	0.92
Specificity	0.88	0.67	0.85
PPV	0.83	0.71	0.85
NPV	0.83	0.85	0.92
MCC	0.65	0.55	0.77
Youden’s Index	0.64	0.54	0.77
*Concordance with Türkiye (average curve)*			
Pearson *r*	–	0.922	0.939
Spearman *ρ*	–	0.921	0.941
Lin’s CCC	–	0.890	0.672
RMSD (pp)	–	4.22	12.48
MAD (pp)	–	3.65	9.36

a Western Asia zone excludes Türkiye. All analyses use the same 10 of 12 seasons (2013/2014–2024/2025, excluding 2020/2021 and 2021/2022 COVID-19 pandemic seasons, and 2019/2020 truncated after week 5). *Source*: ILI-based sentinel surveillance reported to WHO FluNet. Zone positivity = pooled total influenza-positive specimens / total specimens tested, summed across reporting countries per week; 14 Western Asia countries excluding Türkiye and 19 South West Europe countries contributed data (listed in the footnotes to Supplementary Tables S2 and S3). Slope parameter optimized independently per entity by maximizing MCC; all other MEM parameters are package defaults (Supplementary Table S4). Rows under Average Curve Characteristics describe the MEM-modelled average curve, not the observed weekly series. Epidemic and intensity thresholds are upper one-sided confidence limits.

MEM, Moving Epidemic Method; ILI, influenza-like illness; CI, confidence interval; SD, standard deviation; W, epidemiological week; WA, Western Asia; SWE, South West Europe; PPV, positive predictive value; NPV, negative predictive value; MCC, Matthews correlation coefficient; CCC, Lin’s concordance correlation coefficient; RMSD, root mean square deviation; MAD, mean absolute difference; pp., percentage points.

The epidemic threshold was calculated as the upper one-sided limit of the arithmetic mean plus point confidence interval of the pooled pre-epidemic values (i.type.threshold = 5, defined in the package documentation as arithmetic mean and point confidence interval using the standard deviation of the sample; i.tails.threshold = 1; i.level.threshold = 0.95). Intensity thresholds were calculated as the corresponding upper one-sided limits of the geometric mean plus point confidence interval computed on the log scale and back-transformed (i.type.intensity = 6, geometric mean and point confidence interval using the standard deviation of the log-transformed data; i.tails.intensity = 1), evaluated at the 40%, 90%, and 97.5% levels (i.level.intensity = c(0.4, 0.9, 0.975)) to give the medium, high and very high thresholds. In both cases only the upper limit is retained: the package documents the returned pre- and post-epidemic intervals as those whose upper limit constitutes the threshold. No two-sided limits were used for either threshold.

Two-sided intervals were used only for quantities that describe a central estimate rather than a detection boundary. The average curve was constructed from the geometric mean with two-sided 95% confidence intervals (i.type.curve = 2; i.level.curve = 0.95), and the secondary estimators for epidemic start, duration, and percentage used the median with Hettmansperger–Nyblom confidence intervals (i.type.other = 3; i.level.other = 0.95). Confidence intervals were based on *z* values rather than *t* values (i.use.*t* = FALSE, the package default); the implications of this are noted in the Limitations.

### Model performance assessment

Cross-validated goodness-of-fit was assessed iteratively: each season served as test data while the remainder generated thresholds. Pooled sensitivity, specificity, PPV, NPV, percent agreement, MCC, and Youden’s Index were computed. The optimization procedure evaluated slope parameters from 1.0 to 5.0 in increments of 0.1, maximizing MCC; this is the default grid of the mem package (i.detection.values = seq(1, 5, 0.1)). Cross-validation rather than sequential validation was used (i.goodness.method = cross, the package default), so that for each test season the thresholds were derived from all other seasons in the model, both preceding and subsequent [5,7].

### Regional comparison

Türkiye is classified in the WHO Western Asia influenza transmission zone [20]. MEM outputs were compared across three entities – Türkiye, Western Asia excluding Türkiye (WA), and South West Europe (SWE) – each using the same ten seasons and the same ILI-based sentinel indicator. Zone-level positivity rates were calculated as pooled rates: for each epidemiological week of each season, influenza-positive specimens were summed across all reporting countries in the zone and divided by the summed number of specimens tested. Countries reporting no specimens in a given week contributed zero to both numerator and denominator and therefore did not affect that week’s rate, with the consequence that the composition of the pool varies between weeks. Of the 18 countries and territories comprising the Western Asia zone, 14 excluding Türkiye reported sentinel data over the study period and were pooled; Iraq, Oman and Qatar reported none. Of the 24 comprising the South West Europe zone, 19 reported data and were pooled; Andorra, Gibraltar, Liechtenstein, Monaco, and San Marino reported none. The constituent countries of each pool are listed in the footnotes to Supplementary Tables S2 and S3. Each entity was independently parameterized (Türkiye and WA: slope 1.8; SWE: slope 2.3). Concordance was assessed using Pearson’s *r*, Spearman’s rho, Lin’s concordance correlation coefficient (CCC) [33], root mean square deviation (RMSD), and mean absolute difference (MAD). The correlation coefficients and the agreement measures answer different questions, and both are required here. Pearson’s *r* and Spearman’s rho are invariant to location and scale, so two curves of identical shape but very different magnitude will correlate almost perfectly; they therefore assess only whether the curves rise and fall together. Because a central question of this study is whether Türkiye’s epidemic curve operates at the same level as each zone’s, and not merely on the same timetable, correlation alone cannot answer it. Lin’s CCC additionally penalizes departure from the identity line and so captures agreement in shape and level jointly, while RMSD and MAD express the magnitude of disagreement in the original units (percentage points), the form in which a surveillance practitioner would interpret it [33].

### Ethical considerations

This study used aggregated, de-identified, publicly available surveillance data from FluNet. Ethical approval was not required.

## Results

### Descriptive findings

**Figure 1.**
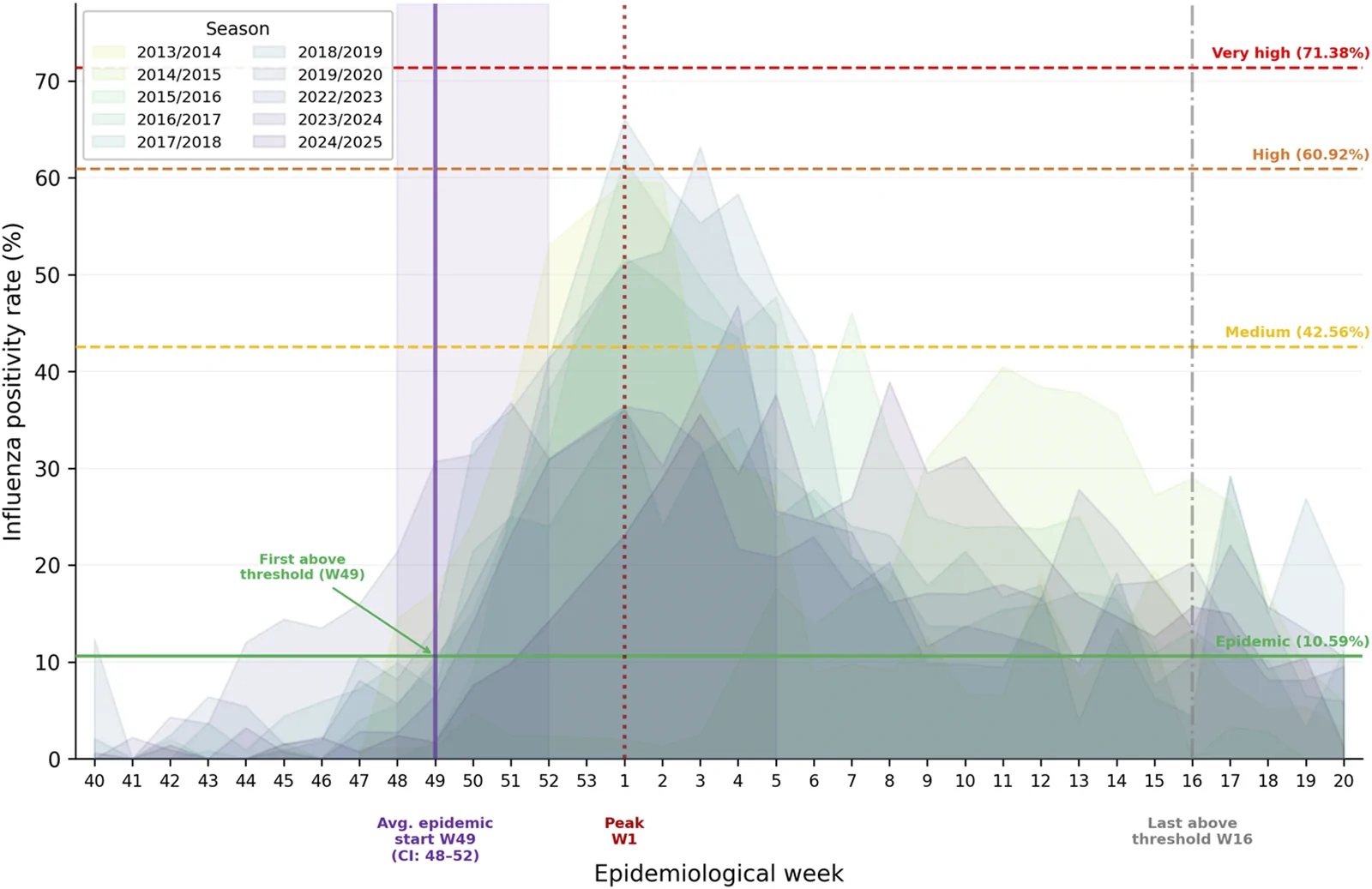
Weekly influenza positivity rates from ILI-based sentinel surveillance, Türkiye, 2013/2014 to 2024/2025 (epidemiological week 40 to week 20). Each line represents one influenza season. The two COVID-19 pandemic seasons (2020/2021 and 2021/2022) were excluded from the MEM analysis but are shown here for completeness; the 2019/2020 season is truncated after week 5. Positivity rate = influenza-positive specimens/specimens tested × 100 (data source: WHO FluNet). Figure 1 long description.

Ten seasons of sentinel data revealed considerable inter-seasonal variability (Supplementary Table S1, Figure 1). The 2018/2019 season demonstrated the highest peak (66.1%, week 1), while 2014/2015 showed an atypically delayed pattern peaking at 40.5% in week 11. The first post-COVID season (2022/2023) exhibited early onset (36.8%, week 51). The 2019/2020 season reached 63.2% in week 3 before truncation. The most recent season (2024/2025) peaked at 38.9% in week 8.

### MEM outputs and model performance

**Figure 2.**
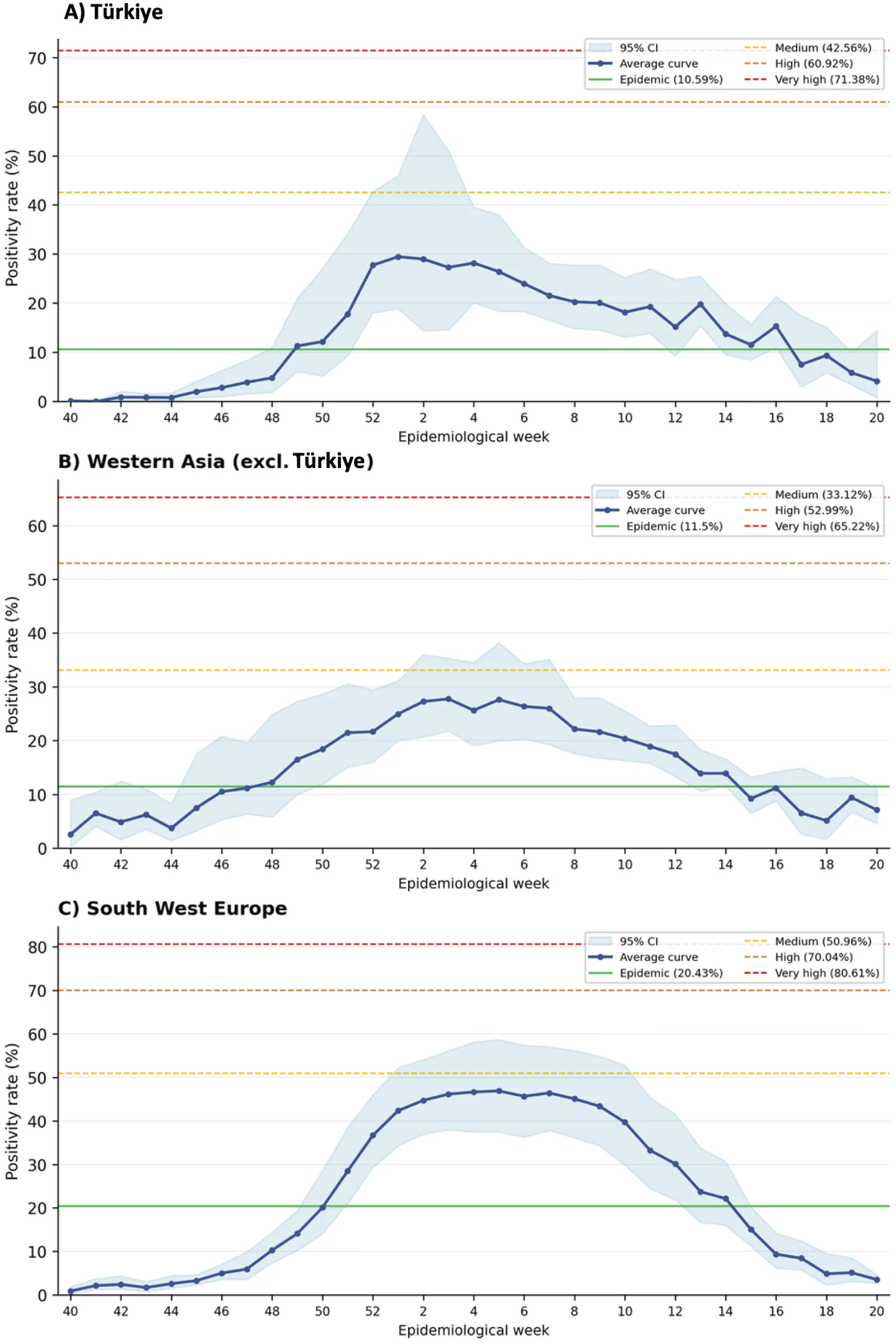
MEM-derived average epidemic curves with 95% confidence intervals and intensity thresholds for influenza positivity rates. Panel A: Türkiye (slope parameter 1.8; ten seasons, 2013/2014–2024/2025, excluding COVID-19 pandemic seasons). Panel B: Western Asia zone excluding Türkiye (slope parameter 1.8; ten seasons). Panel C: South West Europe zone (slope parameter 2.3; ten seasons). The shaded blue area represents the 95% confidence interval around the geometric mean average curve (solid blue line). Horizontal lines indicate the epidemic threshold (green, solid), medium intensity threshold (yellow, dashed), high intensity threshold (orange, dashed), and very high intensity threshold (red, dashed). Zone-level positivity rates were calculated as pooled rates (total positives / total tests) across all reporting countries within each zone per epidemiological week; constituent countries are listed in the footnotes to Supplementary Tables S2 and S3. MEM, Moving Epidemic Method; CI, confidence interval; W, epidemiological week; WA, Western Asia; SWE, South West Europe. Figure 2 long description.

The MEM yielded an epidemic threshold of 10.59% positivity, with intensity thresholds at 42.56% (medium), 60.92% (high), and 71.38% (very high). The average epidemic started at week 49 (95% CI: 48–52), lasting 20.5 weeks and capturing 94.12% of seasonal activity (Table 1). Figure 2A displays the average curve with confidence intervals and thresholds. Cross-validated performance showed sensitivity of 0.77, specificity of 0.88, MCC of 0.65, percent agreement of 0.83, and Youden’s Index of 0.64 (Table 1). The optimization procedure returned slope of 1.8 as the optimal slope parameter for Türkiye, matching the value used in the fitted model. Retrospective application classified the 2018/2019 season as the only one reaching high intensity; 2015/2016 and 2019/2020 reached medium-to-high, while 2014/2015 and 2024/2025 remained low-to-medium.

### Regional comparison

**Figure 3.**
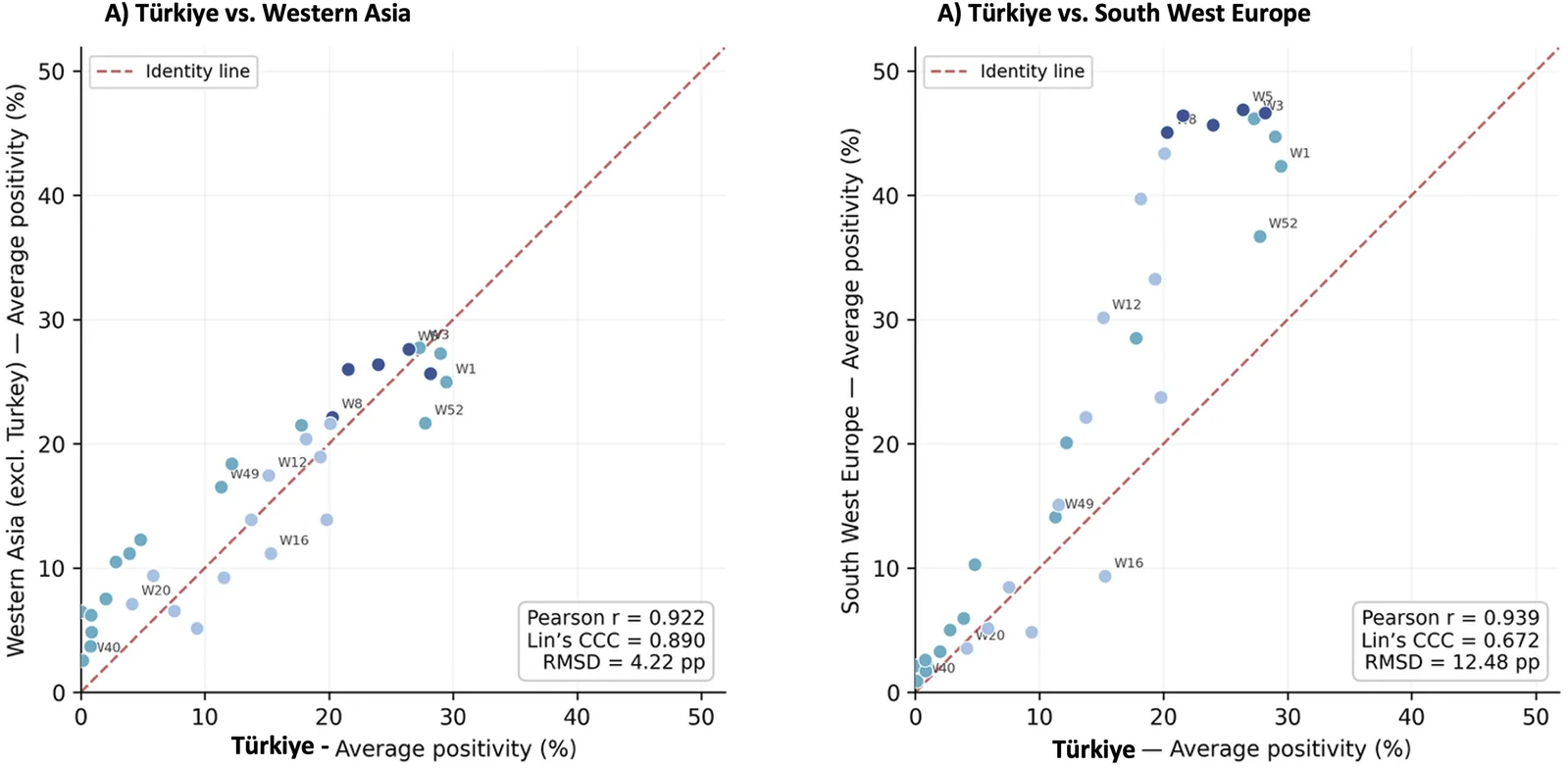
Concordance scatter plots comparing Türkiye’s MEM-derived average epidemic curve with the Western Asia (Panel A) and South West Europe (Panel B) transmission zones. Each point represents one epidemiological week (W40–W20), plotted as Türkiye’s average positivity rate against the corresponding zonal value. The dashed red line indicates perfect agreement (identity line). Points are coloured by epidemic phase: light blue = ascending (W40–W3), dark blue = peak (W4–W8), medium blue = descending (W9–W20). Pearson’s *r*, Lin’s concordance correlation coefficient (CCC), and root mean square deviation (RMSD) are shown for each comparison. In Panel A, points cluster closely around the identity line (CCC = 0.890), indicating strong concordance. In Panel B, points deviate systematically above the identity line, reflecting higher positivity levels in South West Europe despite similar curve shape (Pearson *r* = 0.939), resulting in lower concordance (CCC = 0.672). MEM, Moving Epidemic Method; CCC, Lin’s concordance correlation coefficient; RMSD, root mean square deviation; W, epidemiological week; WA, Western Asia; SWE, South West Europe. Figure 3 long description.

Table 1 presents the complete comparison. Türkiye’s average curve showed high Pearson correlation with both WA (*r* = 0.922) and SWE (*r* = 0.939), but Lin’s CCC diverged markedly: 0.890 for Türkiye–WA versus 0.672 for Türkiye–SWE (Figure 3). This divergence indicates that while Türkiye and SWE share a similar epidemic curve shape, their average curves operate at substantially different absolute levels – points in the Türkiye–WA comparison cluster closely around the identity line, whereas the Türkiye–SWE comparison shows systematic deviation above it (Figure 3). RMSD was 4.22 pp. for Türkiye–WA and 12.48 pp. for Türkiye–SWE. SWE’s average curve peaked at 46.89% (week 5) – nearly 60% higher than Türkiye’s peak of 29.45% (week 1) and WA’s 27.75% (week 3) (Figure 2B,C).

Epidemic thresholds for Türkiye (10.59%) and WA (11.50%) were closely aligned, while SWE was markedly higher (20.43%). Türkiye’s intensity thresholds fell between WA and SWE values across all levels. The MEM-derived epidemic start was week 49 for Türkiye, week 42 for WA (broad CI: 40–1), and week 51 for SWE. Average epidemic duration was longest for WA (24.5 weeks) and shortest for SWE (17.0 weeks), with Türkiye intermediate (20.5 weeks). These findings strongly support Türkiye’s classification within the Western Asia transmission zone.

## Discussion

This study presents the first application of the Moving Epidemic Method to Turkish national sentinel influenza surveillance data, establishing epidemic thresholds and intensity levels for 10 of 12 seasons spanning 2013/2014 to 2024/2025. Within the WHO Western Asia influenza transmission zone, MEM thresholds have previously been published only for Israel by Vega et al. [5], and those were derived from ILI consultation rates for seasons up to 2008/2009. This is therefore, to the best of current knowledge, the first MEM analysis in the zone to derive epidemic and intensity thresholds from virological positivity, and the first to use data from the post-2013 period, filling a gap in the regional surveillance literature.

### Epidemic thresholds in context

The epidemic threshold of 10.59% positivity provides an evidence-based trigger for declaring the onset of seasonal influenza activity in Türkiye. This value is broadly consistent with virological thresholds reported in other European MEM applications: Murray et al. [14] established a 16.7% threshold for primary care virology in Scotland, while the ECDC uses a 10% sentinel positivity threshold at the EU/EEA level to signal the start of seasonal activity [34]. Türkiye’s lower threshold reflects the country’s sentinel system characteristics, including the testing volume, population coverage, and specimen collection practices that influence baseline positivity rates. The model’s specificity (0.88) exceeded its sensitivity (0.77), indicating a conservative threshold that is more likely to miss the earliest epidemic weeks than to generate false alarms. Whether this trade-off is optimal depends on the operational context. In settings where the primary concern is avoiding premature resource mobilization, higher specificity is preferable. However, Türkiye’s sentinel system covers 23 of 81 provinces, with sentinel physicians concentrated in provincial capitals, and routine reporting may itself introduce a lag of 1 to 2 weeks. The cumulative effect of inherent reporting delays and a conservative detection threshold could narrow the window for timely public health response, given that influenza epidemics in Türkiye typically reach peak activity within 4–6 weeks of onset. Future work could evaluate whether a lower slope parameter – accepting reduced specificity in exchange for earlier detection – better serves preparedness needs, particularly for hospital capacity planning and antiviral deployment. Furthermore, the threshold reflects primarily urban sentinel activity; epidemic onset in rural or underserved areas may not be captured at the same positivity level, and subnational threshold calibration should be considered as geographically disaggregated data become available.

### Intensity classification and seasonal severity

The three-tier intensity classification (medium 42.56%, high 60.92%, very high 71.38%) provides a graduated framework for severity assessment. Retrospective application revealed that only the 2018/2019 season reached the high intensity level in Türkiye, with a peak of 66.1%. This season was characterized by A(H1N1)pdm09 and A(H3N2) co-circulation across Europe, with Türkiye among the countries where A(H3N2) predominated, and ILI activity exceeded preceding seasons [35]. The observation that most Turkish seasons remain at low-to-medium intensity aligns with findings from the 28-country European comparison by Vega et al. [6], where most seasons fell within the lower intensity tiers. This suggests that the MEM framework appropriately reserves higher classifications for genuinely exceptional seasons, thereby preserving the signal-to-noise ratio essential for public health communication.

### Regional concordance and transmission zone validation

A key contribution of this study is the empirical evaluation of Türkiye’s WHO transmission zone assignment through quantitative concordance analysis. The divergence between Pearson correlation and Lin’s CCC is particularly informative: while Türkiye’s epidemic curve shape correlates slightly more strongly with SWE (*r* = 0.939) than WA (*r* = 0.922), the level agreement measured by CCC clearly favours WA (0.890 vs. 0.672). This distinction reflects the fundamental difference between correlation (similar shape) and concordance (similar shape at similar levels) – a nuance that Lin emphasized [33] as critical for method comparison studies. The substantially higher mean positivity in SWE (22.32% vs. 13.66% for Türkiye and 15.33% for WA; these are means of the MEM average curves across weeks 40 to 20, not means of the observed weekly series) likely reflects differences in sentinel system design and testing practices across South West European countries rather than genuinely higher influenza transmission, though this cannot be confirmed without standardized population-level incidence data. More selective testing strategies in sentinel systems – where only patients with strong clinical suspicion are tested – mechanically yield higher positivity rates. Cross-zone comparisons of positivity rates are therefore confounded by differences in testing algorithms and case selection criteria, and should not be directly interpreted as reflecting differences in population-level disease burden.

### Western Asia epidemic timing

The MEM-derived average epidemic start of week 42 for the Western Asia zone warrants careful interpretation. This remarkably early start, with a confidence interval spanning 14 weeks (W40–W1), likely reflects the heterogeneity of epidemic timing among constituent countries of the zone. Early-onset seasons in individual countries – particularly during A(H3N2)-dominant years – can pull the zonal median forward substantially. The wide confidence interval indicates that no single “typical” start week adequately characterizes the zone, and country-level MEM analyses within the zone may reveal distinct sub-patterns that are obscured by zonal aggregation. Furthermore, the pooled positivity approach may be disproportionately influenced by countries with the highest testing volumes; in the Western Asia zone a small number of higher-volume reporting countries account for a disproportionate share of all sentinel specimens, and may therefore dominate the zonal epidemic profile.

### The 2014/2015 anomaly and inter-seasonal variability

The 2014/2015 season warrants particular discussion, as it exhibited a markedly delayed epidemic trajectory unlike any other season in the dataset. This atypical pattern, with low positivity during the conventional winter peak and a delayed rise peaking in week 11 at 40.5%, may reflect late-season B/Yamagata dominance [17] or other virological factors. Such outlier seasons can influence MEM threshold calculations, and MEM’s use of the geometric mean for intensity thresholds and the centring algorithm provides some robustness to such outliers. Nevertheless, this underscores the importance of monitoring MEM threshold stability as additional seasons accumulate [7].

### COVID-19 pandemic: Methodological implications

The exclusion of the 2020/2021 and 2021/2022 seasons from the analysis warrants justification. Reported influenza activity fell to historically low levels internationally during the pandemic, in the United States and across Europe [23,24]. In Türkiye the reported positivity for 2020/2021 was likewise at or near zero, but, as set out in section “Data preparation and exclusions”, this most plausibly reflects the pandemic reprioritization of the surveillance and laboratory system rather than a confirmed absence of circulation, and the season is treated as pandemic-affected data rather than as a true low season [28]. In 2021/2022 circulation resumed, but reporting was disrupted, and the compared entities followed sharply divergent epidemic timetables [23,24]. Including such seasons would artificially lower pre-epidemic values, inflate epidemic thresholds, and distort the average curve – effects that were empirically observed when testing 12-season models including pandemic data. The ECDC recommended that MEM thresholds for the 2022/2023 season should be based on pre-pandemic historical data [27], and the UK Health Security Agency similarly excludes the 2019/2020 through 2021/2022 seasons from its MEM baseline calculations. Our approach of truncating 2019/2020 at the end of week 5 and excluding the two fully pandemic-affected seasons is consistent with this emerging consensus.

The post-pandemic landscape introduces additional considerations. The 2022/2023 season, the first full post-COVID season in Türkiye, exhibited an unusually early onset with peak positivity at week 51, potentially reflecting the “immunity debt” phenomenon described globally [25,26]. As post-pandemic seasons continue to accumulate, the stability of MEM thresholds derived from mixed pre- and post-pandemic data will require ongoing monitoring.

### Strengths

This study has several strengths. First, it uses a standardized, internationally endorsed method with freely available software, ensuring reproducibility. Second, the ten-season dataset provides the minimum depth recommended by MEM developers for stable threshold estimation [7]. Third, the regional comparison uses consistent methodology across all three entities – same seasons, same MEM parameters (where appropriate), and pooled positivity rates rather than simple country averages – enabling valid cross-entity comparison. Fourth, the concordance analysis employs Lin’s CCC alongside Pearson and Spearman correlations, providing a more complete assessment of agreement than correlation alone. Fifth, MEM optimization confirmed that the slope parameter (1.8) was independently optimal for Turkish data, providing confidence in the model specification.

### Limitations

Several limitations should be noted. First, the sentinel surveillance system may not be nationally representative, as sentinel sites are typically located in urban areas and may underrepresent rural populations. Second, the positivity rate as an MEM input, while adjusting for testing volume, does not capture absolute disease incidence and is influenced by testing practices and clinical case definitions. Composite indicators combining ILI rates with positivity may offer more interpretable signals [31,36], though the positivity rate remains the most widely available standardized indicator from FluNet. Third, the zone-level pooled positivity approach, while superior to simple country averages, still aggregates heterogeneous surveillance systems and may be dominated by countries with the highest testing volumes. Fourth, with only three full post-pandemic seasons (2022/2023, 2023/2024, and 2024/2025) in the dataset, the influence of pandemic disruption on threshold stability cannot yet be fully assessed. Fifth, the MEM’s odds transformation, recommended for percentage-bounded data in the technical documentation [7], was not applied; sensitivity analyses using this transformation may be warranted. Sixth, only ILI-based sentinel data were analysed; SARI surveillance in Türkiye operates in 11 hospitals across 8 of the 81 provinces and yields a median of 88 specimens per season (IQR, 62–119), which is too sparse a series for stable threshold estimation; MEM applied to SARI data addresses a different question and remains a priority for future work [30,37]. Finally, ECDC and the WHO Regional Office for Europe now recommend year-round integrated respiratory virus surveillance, and Türkiye has made this transition, but off-season sampling volumes remain low: in the 7 weeks following the end of the most recent influenza season Türkiye reported a mean of 19.4 specimens per week, against a sentinel system sizing target of the order of 250 specimens per week [30]. Positivity computed on denominators of this size is volatile, and including such weeks would risk distorting the pre-epidemic distribution from which the epidemic threshold is derived. The thresholds reported here therefore describe the conventional week 40 to week 20 season and are not applicable to out-of-season signal detection.

### Implications for surveillance practice

These findings have direct operational implications for Türkiye’s influenza surveillance programme. The established thresholds can be integrated into real-time monitoring dashboards to provide evidence-based weekly severity assessments, inform clinical guidance and resource allocation during epidemic periods, and contribute to Türkiye’s reporting obligations under the WHO PISA framework [4]. In practice, exceedance of the 10.59% epidemic threshold would signal the start of the influenza season, triggering enhanced reporting and clinical preparedness. Weekly assessment against intensity thresholds would provide graduated severity signals to inform hospital capacity planning, antiviral deployment, and public communication. Integration with Türkiye’s existing HSYS platform would enable near-real-time threshold monitoring at the national level. The strong concordance with the Western Asia zone provides empirical validation of Türkiye’s transmission zone assignment, supporting the use of zone-level information for vaccine timing recommendations and comparative surveillance across the zone.

### Future directions

Several research priorities emerge from this analysis: (1) applying MEM to ILI consultation rates and SARI hospitalization data as complementary indicators, which would enable triangulation across surveillance streams; (2) evaluating the composite indicator (ILI rate × positivity rate) recommended by recent methodological work [31,36] for improved interpretability; (3) performing age-stratified MEM analyses, as epidemic thresholds may differ substantially between paediatric and elderly populations [9]; (4) extending the regional comparison to additional WHO transmission zones to further characterize Türkiye’s epidemiological position; and (5) monitoring threshold stability annually as post-pandemic seasons accumulate and the balance between pre- and post-pandemic data in the model shifts.

## Conclusion

This study establishes, for the first time, MEM-derived epidemic thresholds and intensity levels for influenza in Türkiye and for the WHO Western Asia transmission zone based on virological positivity data. The epidemic threshold of 10.59% positivity and the graduated intensity classification offer an objective, reproducible framework for real-time influenza surveillance. The concordance analysis indicates that Türkiye’s influenza dynamics are more closely aligned with the Western Asia zone than with South West Europe, consistent with its current WHO transmission zone classification. These thresholds should be updated annually as new seasons accumulate and should be incorporated into Türkiye’s national influenza surveillance operations and WHO PISA reporting.

## Supporting information

10.1017/S095026882610209X.sm001 Ontas supplementary material Ontas supplementary material

## Data Availability

The sentinel influenza surveillance data used in this study are publicly available through the WHO Global Influenza Programme FluNet platform (https://www.who.int/tools/flunet). Zone-level aggregated weekly positivity rates and MEM outputs are provided in the manuscript and supplementary material. The analysis can be reproduced with the freely available MEM web application (memapp), described in Supplementary Note S1.

## Supplementary material

The supplementary material for this article can be found at http://doi.org/10.1017/S095026882610209X.

## Long descriptions

### Table 1. Long description

Comparative table for Türkiye, Western Asia excluding Türkiye, and South West Europe. All models use 10 seasons. Optimized slope parameters are 1.8, 1.8 and 2.3, respectively. Average epidemic start is week 49 (CI 48–52), week 42 (CI 40–1), and week 51 (CI 47–52); duration is 20.5, 24.5 and 17.0 weeks; epidemic percentages are 94.12%, 90.95% and 89.11%. Epidemic thresholds are 10.59%, 11.50% and 20.43%; post-epidemic thresholds are 22.04%, 14.09% and 18.61%. Medium, high and very-high intensity thresholds are 42.56%, 60.92%, 71.38% for Türkiye; 33.12%, 52.99%, 65.22% for Western Asia; and 50.96%, 70.04%, 80.61% for South West Europe. Peak positivity is 29.45%, 27.75% and 46.89%, occurring in weeks 1, 3 and 5. The average curves are above threshold for 20, 19 and 16 weeks, with mean positivity 13.66%, 15.33% and 22.32%. Model performance is sensitivity 0.77/0.88/0.92, specificity 0.88/0.67/0.85, PPV 0.83/0.71/0.85, NPV 0.83/0.85/0.92, MCC 0.65/0.55/0.77, and Youden’s index 0.64/0.54/0.77. Concordance with Türkiye is Pearson r 0.922 and 0.939, Spearman rho 0.921 and 0.941, Lin’s CCC 0.890 and 0.672, RMSD 4.22 and 12.48 percentage points, and MAD 3.65 and 9.36 percentage points for Western Asia and South West Europe, respectively.

Navigate back to Table 1..

### Figure 1 Long description

Line chart of weekly influenza positivity rates in Türkiye from epidemiological week 40 through week 20 for 10 analysed seasons (2013/2014–2024/2025). Each line represents one season. The 2020/2021 and 2021/2022 pandemic seasons are shown but were excluded from MEM analysis; 2019/2020 ends after week 5. Positivity generally rises from late autumn, peaks around winter, and declines in spring, but timing and magnitude vary substantially. The 2018/2019 season has the highest observed peak, 66.1% in week 1. The 2014/2015 season is unusually delayed, peaking at 40.5% in week 11. The truncated 2019/2020 season reaches 63.2% in week 3. The first post-COVID season, 2022/2023, shows an early peak of 36.8% in week 51. The 2024/2025 season peaks at 38.9% in week 8.

Navigate back to Figure 1.

### Figure 2 Long description

Three-panel line chart showing MEM-derived average influenza positivity curves with 95% confidence intervals and horizontal epidemic and intensity thresholds. Panel A, Türkiye: the average curve first exceeds the 10.59% epidemic threshold at week 49, peaks at 29.45% in week 1, and remains above the threshold through week 16. Medium, high and very-high intensity thresholds are 42.56%, 60.92% and 71.38%, respectively. Panel B, Western Asia excluding Türkiye: the curve starts above the 11.50% threshold at week 48, peaks at 27.75% in week 3, and remains above threshold through week 14; intensity thresholds are 33.12%, 52.99% and 65.22%. Panel C, South West Europe: the curve starts above the 20.43% threshold at week 51, peaks at 46.89% in week 5, and remains above threshold through week 14; intensity thresholds are 50.96%, 70.04% and 80.61%. The shaded bands show 95% confidence intervals.

Navigate back to Figure 2.

### Figure 3 Long description

Two scatter plots compare Türkiye’s MEM-derived average influenza positivity curve with corresponding weekly values for Western Asia excluding Türkiye and South West Europe. Each point represents an epidemiological week from week 40 to week 20; the dashed diagonal line represents perfect agreement. For Türkiye versus Western Asia, points cluster relatively close to the identity line, with Pearson r = 0.922, Lin’s concordance correlation coefficient (CCC) = 0.890, and RMSD = 4.22 percentage points. For Türkiye versus South West Europe, the points lie systematically above the identity line, indicating higher positivity in South West Europe despite similar temporal shape; Pearson r = 0.939, CCC = 0.672, and RMSD = 12.48 percentage points. Points are coloured by ascending, peak, and descending epidemic phases.

Navigate back to Figure 3.
