# Supplementary material for: Establishing influenza epidemic thresholds and intensity levels in Türkiye using the moving epidemic method and assessing concordance with WHO transmission zones: A sentinel surveillance analysis of ten seasons, 2013–2025
Source: Epidemiol Infect. 2026 Oct 15;154:e129. doi: 10.1017/S095026882610209X (PMC13749099; doi:10.1017/S095026882610209X)
Supplement: Supplementary file 1 [file S095026882610209Xsup001.docx]

**Establishing Influenza Epidemic Thresholds and Intensity Levels in Türkiye Using the Moving Epidemic Method and Assessing Concordance with WHO Transmission Zones: A Sentinel Surveillance Analysis of Ten Seasons, 2013–2025**

*Epidemiology and Infection*

Eray Ontas

<https://doi.org/10.1017/S095026882610209X>

# **SUPPLEMENTS**

**Supplement Table S1.** Weekly influenza positivity rates (%) from sentinel surveillance, Türkiye, 2013/2014–2024/2025 (Week 40–Week 20).

**Supplement Table S2.** Weekly influenza positivity rates (%), Western Asia zone (excl. Türkiye), pooled across reporting countries, 2013/2014–2024/2025.

**Supplement Table S3.** Weekly influenza positivity rates (%), South West Europe zone, pooled across reporting countries, 2013/2014–2024/2025.

**Supplement Table S4.** MEM parameterization: selected values and rationale.

**Supplement Figure S1.** Seasonal influenza positivity rates with MEM-derived epidemic and intensity thresholds, Türkiye, 2013/2014–2024/2025 (10 seasons, COVID-19 seasons excluded).

**Supplement Note S1.** Software and reproducibility: the Moving Epidemic Method Shiny web application (memapp).

| **Supplement Table S1. Weekly influenza positivity rates (%) from sentinel surveillance, Türkiye, 2013/2014–2024/2025 (Week 40–Week 20).** | | | | | | | | | | | | |
| --- | --- | --- | --- | --- | --- | --- | --- | --- | --- | --- | --- | --- |
| “–” indicates a week with no reported value. Shaded columns (2020/2021, 2021/2022) were excluded from the MEM analysis and are shown for reference only; see Methods Section 2.2 for the season-specific rationale. During these two pandemic seasons the reported values ran at or near zero across all entities and are regarded as pandemic-affected and unreliable, reflecting reduced and reprioritised sentinel testing rather than confirmed low circulation. The 2019/2020 season is truncated after week 5. Indicator: weekly influenza positivity rate = influenza-positive specimens / specimens tested × 100, from ILI-based sentinel surveillance reported to WHO FluNet. ILI = influenza-like illness; MEM = Moving Epidemic Method; W = epidemiological week. | | | | | | | | | | | | |
|  |  |  |  |  |  |  |  |  |  |  |  |  |
| **Week No** | **2013/2014** | **2014/2015** | **2015/2016** | **2016/2017** | **2017/2018** | **2018/2019** | **2019/2020** | **2020/2021** | **2021/2022** | **2022/2023** | **2023/2024** | **2024/2025** |
| **40** | 0.0 | – | 0.0 | 0.0 | 0.0 | 2.1 | 12.3 | 0.0 | 0.0 | 0.0 | 0.0 | 0.6 |
| **41** | 0.0 | – | 0.0 | 0.0 | 0.0 | 0.0 | 0.0 | 0.0 | 0.0 | 0.0 | 2.2 | 0.0 |
| **42** | – | – | 2.0 | 0.0 | 0.0 | 0.0 | 2.4 | – | 0.0 | 4.3 | 0.9 | 1.4 |
| **43** | 0.0 | – | 0.0 | 0.8 | 3.7 | 0.0 | 6.4 | 0.0 | 0.0 | 3.6 | 0.0 | 0.0 |
| **44** | 0.0 | – | 0.0 | 0.0 | 0.9 | 0.0 | 5.4 | 0.0 | 0.0 | 12.0 | 3.2 | 0.0 |
| **45** | 0.9 | – | 1.6 | 0.0 | 4.4 | 0.9 | 1.5 | 0.0 | 0.0 | 14.4 | 0.7 | 1.5 |
| **46** | 0.0 | – | 0.0 | 0.0 | 5.9 | 1.8 | 2.0 | 0.0 | 8.2 | 13.5 | 0.0 | 2.2 |
| **47** | 0.0 | 1.1 | 0.0 | 4.0 | 7.2 | 10.6 | 8.1 | 0.0 | 4.2 | 16.0 | 2.8 | 0.7 |
| **48** | 14.5 | 1.1 | 0.0 | 5.6 | 9.9 | 8.2 | 5.7 | 0.0 | 13.0 | 21.4 | 2.7 | 2.4 |
| **49** | 17.3 | 1.2 | 1.8 | 10.6 | 7.2 | 13.7 | 9.9 | 0.0 | 37.7 | 30.7 | 6.5 | 1.7 |
| **50** | 24.6 | 4.8 | 9.0 | 15.2 | 21.5 | 32.8 | 17.5 | 0.0 | 29.2 | 31.4 | 14.1 | 7.6 |
| **51** | 36.2 | 2.4 | 25.9 | 24.7 | 25.0 | 36.0 | 25.3 | 0.0 | 31.8 | 36.8 | 23.1 | 9.9 |
| **52** | 53.0 | 2.3 | 32.5 | 38.1 | 24.0 | 41.4 | 41.3 | 0.0 | 43.5 | 31.0 | 30.9 | 14.2 |
| **53** | – | – | 48.9 | – | – | – | – | 0.0 | – | – | – | – |
| **1** | 59.7 | 2.0 | 61.9 | 51.8 | 36.4 | 66.1 | 51.2 | 0.0 | 39.8 | 36.4 | 36.0 | 23.1 |
| **2** | 59.4 | 1.3 | 56.1 | 49.2 | 24.0 | 60.0 | 52.4 | 0.0 | 36.0 | 35.7 | 30.2 | 29.0 |
| **3** | 37.6 | 2.5 | 49.7 | 45.4 | 31.4 | 55.3 | 63.2 | 0.0 | 34.0 | 32.4 | – | 35.6 |
| **4** | 30.6 | 9.9 | 44.3 | 43.5 | 34.2 | 58.3 | 50.0 | 0.0 | 8.6 | 21.7 | 46.8 | 29.4 |
| **5** | 28.2 | 17.6 | 47.7 | 30.1 | 24.8 | 48.7 | 44.8 | 0.0 | 2.0 | 20.8 | 25.6 | 37.6 |
| **6** | 9.0 | 13.8 | 33.8 | 26.9 | 27.8 | 41.9 | – | 0.0 | 1.9 | 22.9 | 24.5 | 24.7 |
| **7** | 9.8 | 16.8 | 46.1 | 20.7 | 24.0 | 20.9 | – | 0.0 | 5.1 | 17.5 | 23.4 | 26.9 |
| **8** | 9.1 | 18.4 | 33.1 | 19.6 | 23.1 | 17.2 | – | 0.0 | 1.9 | 20.3 | 16.1 | 38.9 |
| **9** | 12.5 | 31.1 | 25.0 | 13.7 | 17.9 | 9.8 | – | 0.0 | 2.1 | 11.6 | 17.1 | 29.5 |
| **10** | 6.7 | 35.5 | 23.9 | 13.6 | 21.4 | 9.8 | – | 0.0 | 0.0 | 13.7 | 17.0 | 31.2 |
| **11** | 6.5 | 40.5 | 24.0 | 15.4 | 16.7 | 9.4 | – | 0.0 | 0.0 | 12.8 | 18.0 | 25.9 |
| **12** | 18.9 | 38.4 | 23.7 | 15.9 | 17.9 | 16.7 | – | 2.7 | 0.0 | 11.7 | 16.5 | 21.4 |
| **13** | 8.0 | 37.8 | 25.0 | 17.2 | 3.8 | 9.6 | – | 0.0 | 4.7 | 9.9 | 27.8 | 16.7 |
| **14** | 11.7 | 35.6 | 17.3 | 16.5 | 13.5 | 19.3 | – | 0.0 | 3.1 | 18.0 | 23.6 | – |
| **15** | 19.6 | 27.2 | 11.9 | 10.9 | 6.3 | 7.6 | – | 0.0 | 4.8 | 18.3 | – | 12.6 |
| **16** | 13.0 | 29.0 | 0.0 | 13.3 | 4.4 | 10.6 | – | 0.0 | 0.0 | 20.3 | 13.5 | 15.7 |
| **17** | 7.7 | 26.5 | 3.3 | 10.4 | 29.2 | 29.2 | – | 0.0 | 0.0 | 13.2 | 22.1 | 15.0 |
| **18** | 5.1 | 16.9 | 2.8 | 10.2 | 15.1 | 15.1 | – | 0.0 | 0.0 | 8.1 | 15.7 | 9.3 |
| **19** | 5.4 | 9.4 | 0.0 | 3.2 | 6.5 | 26.9 | – | – | 3.8 | 8.1 | 13.3 | 10.4 |
| **20** | 2.9 | 5.8 | 0.0 | 11.1 | 5.9 | 17.9 | – | 0.0 | 0.0 | 9.5 | 10.5 | 1.2 |

| **Supplement Table S2. Weekly influenza positivity rates (%), Western Asia (excl. Türkiye), pooled across reporting countries, 2013/2014–2024/2025.** | | | | | | | | | | | | |
| --- | --- | --- | --- | --- | --- | --- | --- | --- | --- | --- | --- | --- |
| Pooled rate = total influenza-positive specimens / total specimens tested, summed across all reporting countries of the zone for each week. Countries reporting no specimens in a given week contribute zero to both numerator and denominator, so the composition of the pool varies between weeks. The WHO Western Asia influenza transmission zone (version dated 24 September 2025) comprises 18 countries and territories. Excluding Türkiye, the following 14 reported ILI-based sentinel data over the study period and were pooled: Armenia, Azerbaijan, Bahrain, Cyprus, Georgia, Israel, Jordan, Kuwait, Lebanon, the occupied Palestinian territory including east Jerusalem, Saudi Arabia, the Syrian Arab Republic, the United Arab Emirates and Yemen. Three zone members — Iraq, Oman and Qatar — reported no data and are therefore not represented. Shaded columns (2020/2021, 2021/2022) were excluded from the MEM analysis and are shown for reference only. ILI = influenza-like illness; MEM = Moving Epidemic Method; W = epidemiological week. | | | | | | | | | | | | |
|  |  |  |  |  |  |  |  |  |  |  |  |  |
| **Week No** | **2013/2014** | **2014/2015** | **2015/2016** | **2016/2017** | **2017/2018** | **2018/2019** | **2019/2020** | **2020/2021** | **2021/2022** | **2022/2023** | **2023/2024** | **2024/2025** |
| **40** | 0.0 | 0.0 | 2.7 | 0.0 | 9.9 | 8.2 | 13.7 | 1.8 | 1.2 | 20.9 | 17.3 | 5.0 |
| **41** | 0.0 | 0.0 | 7.7 | 0.0 | 13.1 | 13.0 | 17.4 | 0.0 | 1.0 | 24.9 | 23.0 | 6.7 |
| **42** | 5.9 | 0.0 | 8.2 | 2.6 | 14.6 | 16.7 | 19.4 | 0.0 | 1.1 | 21.6 | 26.0 | 9.6 |
| **43** | 9.2 | 0.0 | 4.9 | 0.0 | 11.6 | 15.5 | 26.6 | 0.0 | 1.5 | 24.4 | 27.1 | 11.3 |
| **44** | 2.8 | 0.0 | 4.5 | 1.9 | 17.3 | 11.4 | 27.3 | 1.1 | 5.0 | 25.1 | 26.5 | 12.0 |
| **45** | 3.2 | 5.0 | 6.0 | 3.9 | 19.7 | 16.6 | 27.1 | 0.4 | 6.5 | 25.1 | 19.5 | 14.8 |
| **46** | 0.0 | 6.8 | 1.9 | 24.1 | 20.3 | 18.6 | 30.7 | 1.6 | 9.5 | 22.9 | 19.0 | 16.6 |
| **47** | 0.6 | 8.8 | 3.8 | 30.4 | 22.5 | 21.0 | 27.2 | 1.1 | 6.0 | 19.5 | 18.4 | 16.6 |
| **48** | 1.3 | 2.0 | 4.4 | 18.0 | 22.8 | 12.8 | 28.2 | 0.6 | 7.6 | 17.4 | 17.2 | 16.6 |
| **49** | 2.2 | 0.7 | 3.5 | 34.5 | 22.3 | 18.7 | 33.5 | 0.6 | 8.5 | 17.7 | 16.0 | 16.1 |
| **50** | 0.0 | 2.4 | 7.2 | 45.7 | 21.2 | 20.8 | 29.9 | 0.3 | 14.5 | 16.7 | 16.0 | 18.0 |
| **51** | 2.8 | 3.8 | 14.9 | 58.8 | 27.6 | 20.8 | 32.5 | 0.3 | 19.4 | 14.4 | 15.0 | 18.7 |
| **52** | 3.6 | 4.8 | 19.0 | 44.5 | 29.1 | 29.3 | 34.0 | 0.5 | 23.0 | 16.3 | 13.8 | 20.3 |
| **53** | – | – | 40.7 | – | – | – | – | 0.7 | – | – | – | – |
| **1** | 5.7 | 6.1 | 42.0 | 37.8 | 37.9 | 27.9 | 40.1 | 0.5 | 15.0 | 16.5 | 13.8 | 17.7 |
| **2** | 8.3 | 10.8 | 42.1 | 27.4 | 32.0 | 29.2 | 35.7 | 1.6 | 7.8 | 16.4 | 10.4 | 22.1 |
| **3** | 16.2 | 20.8 | 49.6 | 34.3 | 28.5 | 35.4 | 29.7 | 0.9 | 4.7 | 16.4 | 9.6 | 20.8 |
| **4** | 14.9 | 13.9 | 47.7 | 21.4 | 28.0 | 33.0 | 33.5 | 0.8 | 1.7 | 15.7 | 11.0 | 20.0 |
| **5** | 32.1 | 40.5 | 39.8 | 15.2 | 24.0 | 30.6 | 27.2 | 0.9 | 2.3 | 13.6 | 10.8 | 23.0 |
| **6** | 30.0 | 45.6 | 50.4 | 13.6 | 26.8 | 29.5 | 24.7 | 1.1 | 0.2 | 14.8 | 13.8 | 21.9 |
| **7** | 51.9 | 45.2 | 38.5 | 18.0 | 29.2 | 28.2 | 20.2 | 0.6 | 0.5 | 16.6 | 11.0 | 20.1 |
| **8** | 37.7 | 58.4 | 39.0 | 19.0 | 26.0 | 32.4 | 19.1 | 1.1 | 1.6 | 12.4 | 10.4 | 16.8 |
| **9** | 46.3 | 50.0 | 20.8 | 17.9 | 22.1 | 23.0 | 21.7 | 0.3 | 2.7 | 11.4 | 10.9 | 13.6 |
| **10** | 29.4 | 45.9 | 24.6 | 12.4 | 20.6 | 23.2 | 10.5 | 0.3 | 1.3 | 11.6 | 8.2 | 13.5 |
| **11** | 42.0 | 44.6 | 16.0 | 19.4 | 17.3 | 24.3 | 6.5 | 0.3 | 1.1 | 11.1 | 10.0 | 13.5 |
| **12** | 38.8 | 25.6 | 13.6 | 14.9 | 14.7 | 23.0 | 1.2 | 0.0 | 7.0 | 9.4 | 8.0 | 10.5 |
| **13** | 32.5 | 23.0 | 34.4 | 14.1 | 11.1 | 25.0 | 0.5 | 0.1 | 4.8 | 9.3 | 5.5 | 7.4 |
| **14** | 31.8 | 25.2 | 21.4 | 18.4 | 16.5 | 19.1 | 1.2 | 0.3 | 1.2 | 7.5 | 7.3 | 7.1 |
| **15** | 10.3 | 21.1 | 10.3 | 14.3 | 13.7 | 22.2 | 20.7 | 0.0 | 3.0 | 5.0 | 4.1 | 6.2 |
| **16** | 20.8 | 8.6 | 4.7 | 10.8 | 15.7 | 16.6 | 0.0 | 0.0 | 3.3 | 8.7 | 4.8 | 5.4 |
| **17** | 7.8 | 5.5 | 5.5 | 12.7 | 15.9 | 16.5 | 0.0 | 0.2 | 2.9 | 5.4 | 5.2 | 6.0 |
| **18** | 12.8 | 11.4 | 1.2 | 20.2 | 10.7 | 10.8 | 0.0 | 0.3 | 3.7 | 4.6 | 3.8 | 5.0 |
| **19** | 0.0 | 3.0 | 0.0 | 20.0 | 13.4 | 13.1 | 0.0 | 0.0 | 4.8 | 6.4 | 4.0 | 6.1 |
| **20** | 0.0 | 16.7 | 3.2 | 16.9 | 7.8 | 11.1 | 0.0 | 0.0 | 4.9 | 7.3 | 3.1 | 4.1 |

| **Supplement Table S3. Weekly influenza positivity rates (%), South West Europe, pooled across reporting countries, 2013/2014–2024/2025.** | | | | | | | | | | | | |
| --- | --- | --- | --- | --- | --- | --- | --- | --- | --- | --- | --- | --- |
| Pooled rate = total influenza-positive specimens / total specimens tested, summed across all reporting countries of the zone for each week, on the same basis as Supplement Table S2. The WHO South West Europe influenza transmission zone (version dated 24 September 2025) comprises 24 countries and territories, of which the following 19 reported ILI-based sentinel data over the study period and were pooled: Albania, Austria, Belgium, Bosnia and Herzegovina, Croatia, France, Germany, Greece, Italy, Luxembourg, Malta, Montenegro, the Netherlands (Kingdom of the), North Macedonia, Portugal, Serbia, Slovenia, Spain and Switzerland. Five zone members — Andorra, Gibraltar (the United Kingdom), Liechtenstein, Monaco and San Marino — reported no data; all are microstates or dependent territories without independent sentinel reporting to FluNet. Shaded columns (2020/2021, 2021/2022) were excluded from the MEM analysis and are shown for reference only. ILI = influenza-like illness; MEM = Moving Epidemic Method; W = epidemiological week. | | | | | | | | | | | | |
|  |  |  |  |  |  |  |  |  |  |  |  |  |
| **Week No** | **2013/2014** | **2014/2015** | **2015/2016** | **2016/2017** | **2017/2018** | **2018/2019** | **2019/2020** | **2020/2021** | **2021/2022** | **2022/2023** | **2023/2024** | **2024/2025** |
| **40** | 2.8 | 3.4 | 0.0 | 0.0 | 2.7 | 0.8 | 2.8 | 0.0 | 0.3 | 7.7 | 1.6 | 1.3 |
| **41** | 0.8 | 2.9 | 1.0 | 3.7 | 1.9 | 0.0 | 2.3 | 0.0 | 0.5 | 8.5 | 1.3 | 1.5 |
| **42** | 0.0 | 1.1 | 1.5 | 3.4 | 4.7 | 2.6 | 3.1 | 0.0 | 0.5 | 9.3 | 1.2 | 1.8 |
| **43** | 0.7 | 4.0 | 0.9 | 4.7 | 1.9 | 1.0 | 1.0 | 0.0 | 0.3 | 10.7 | 2.1 | 1.5 |
| **44** | 0.8 | 4.7 | 3.1 | 5.4 | 1.6 | 1.3 | 3.8 | 0.0 | 0.4 | 9.9 | 2.1 | 2.5 |
| **45** | 0.6 | 3.0 | 1.5 | 10.2 | 5.1 | 2.1 | 6.3 | 0.0 | 0.6 | 13.7 | 2.0 | 2.7 |
| **46** | 0.4 | 3.4 | 6.6 | 15.5 | 4.9 | 2.7 | 7.7 | 0.0 | 1.0 | 16.9 | 3.6 | 3.5 |
| **47** | 1.4 | 3.0 | 3.8 | 22.9 | 11.2 | 4.1 | 10.2 | 0.0 | 1.2 | 20.2 | 5.2 | 3.3 |
| **48** | 4.2 | 9.9 | 2.4 | 24.7 | 22.0 | 6.5 | 18.6 | 0.0 | 2.1 | 27.3 | 7.5 | 6.8 |
| **49** | 5.3 | 10.1 | 6.0 | 38.3 | 21.9 | 10.5 | 18.7 | 0.2 | 3.2 | 34.5 | 9.6 | 8.7 |
| **50** | 12.4 | 13.8 | 5.2 | 48.7 | 40.9 | 10.6 | 24.8 | 0.0 | 3.9 | 38.4 | 18.8 | 11.9 |
| **51** | 17.4 | 27.3 | 11.5 | 55.7 | 52.2 | 17.6 | 35.6 | 0.0 | 3.9 | 41.3 | 25.1 | 19.5 |
| **52** | 26.9 | 36.9 | 16.3 | 58.7 | 65.7 | 36.3 | 40.2 | 0.2 | 7.7 | 32.1 | 37.3 | 27.9 |
| **53** | – | – | 14.2 | – | – | – | – | 0.0 | – | – | – | – |
| **1** | 38.1 | 37.3 | 22.0 | 54.1 | 57.1 | 35.0 | 40.5 | 0.0 | 6.4 | 25.2 | 33.8 | 33.1 |
| **2** | 45.3 | 45.2 | 27.4 | 56.2 | 60.1 | 44.7 | 43.1 | 0.2 | 4.6 | 20.5 | 32.7 | 40.9 |
| **3** | 51.4 | 56.9 | 41.0 | 55.7 | 62.0 | 51.9 | 51.2 | 0.0 | 5.4 | 20.2 | 37.7 | 42.8 |
| **4** | 49.1 | 58.1 | 48.8 | 58.6 | 64.3 | 60.2 | 57.7 | 0.0 | 6.6 | 20.5 | 39.4 | 49.1 |
| **5** | 46.0 | 60.6 | 53.5 | 54.2 | 62.5 | 63.8 | 64.3 | 0.0 | 9.1 | 26.0 | 33.1 | 50.3 |
| **6** | 39.3 | 61.7 | 58.7 | 49.4 | 60.4 | 62.2 | 61.5 | 0.1 | 10.0 | 28.4 | 32.3 | 44.9 |
| **7** | 37.0 | 65.0 | 57.2 | 43.3 | 63.6 | 61.5 | 59.9 | 0.1 | 8.9 | 31.2 | 24.3 | 42.9 |
| **8** | 36.0 | 65.0 | 59.2 | 35.9 | 63.9 | 57.6 | 55.2 | 0.0 | 10.5 | 31.5 | 20.0 | 40.2 |
| **9** | 31.6 | 58.5 | 60.1 | 29.3 | 61.1 | 52.1 | 49.6 | 0.1 | 16.2 | 27.7 | 13.1 | 36.3 |
| **10** | 32.6 | 56.3 | 62.0 | 23.2 | 61.9 | 51.0 | 42.9 | 0.0 | 27.5 | 29.2 | 11.9 | 33.0 |
| **11** | 32.4 | 53.7 | 63.9 | 18.0 | 56.1 | 45.1 | 32.7 | 0.0 | 27.5 | 28.0 | 9.1 | 27.6 |
| **12** | 34.8 | 53.9 | 65.3 | 17.9 | 48.6 | 36.9 | 16.5 | 0.0 | 31.5 | 25.0 | 7.7 | 20.1 |
| **13** | 19.6 | 44.4 | 60.3 | 5.4 | 44.0 | 32.3 | 4.9 | 0.2 | 32.0 | 16.5 | 8.8 | 17.3 |
| **14** | 28.0 | 46.2 | 51.3 | 16.1 | 32.1 | 28.1 | 0.8 | 0.0 | 26.4 | 14.7 | 6.7 | 12.0 |
| **15** | 12.4 | 34.6 | 47.0 | 14.8 | 22.8 | 17.7 | 1.6 | 0.0 | 28.2 | 11.1 | 5.3 | 8.7 |
| **16** | 19.5 | 43.9 | 35.4 | 14.6 | 14.2 | 10.6 | 0.0 | 0.0 | 18.8 | 8.0 | 5.8 | 7.4 |
| **17** | 20.3 | 23.9 | 24.8 | 2.9 | 10.8 | 6.0 | 0.0 | 0.0 | 14.8 | 7.4 | 4.4 | 4.3 |
| **18** | 7.8 | 28.6 | 28.7 | 0.0 | 10.3 | 4.3 | 0.0 | 0.2 | 12.4 | 4.9 | 5.1 | 3.1 |
| **19** | 7.1 | 17.6 | 19.7 | 4.9 | 3.1 | 3.9 | 0.0 | 0.0 | 13.2 | 4.1 | 3.6 | 2.5 |
| **20** | 7.0 | 0.0 | 11.5 | 3.6 | 4.5 | 4.3 | 0.0 | 0.0 | 10.0 | 2.6 | 2.5 | 2.3 |

| **Supplement Table S4. MEM parameterization: selected values and rationale for the Türkiye sentinel influenza surveillance analysis.** | | |
| --- | --- | --- |
| Parameters apply to all three entities (Türkiye, Western Asia excl. Türkiye, South West Europe) unless otherwise noted. Values are given with the corresponding mem package argument so that the specification can be verified directly against the package documentation (Lozano 2023) and the MEM Technical Manual (Lozano and Vega 2020). Of the parameters governing the model, only the slope parameter was chosen empirically; every other value is the mem package default. | | |
| **Parameter** | **Value** | **Rationale** |
| ***General*** | | |
| **Maximum seasons** | 10 (i.seasons = 10; package default) | Ten seasons is the depth MEM developers recommend and the point at which the trade-off turns: Vega et al. (2013) note that more than ten seasons may improve precision but makes the model vulnerable to bias from secular trends in the surveillance system, while fewer reduces threshold stability. |
| **Seasons used (Türkiye)** | 2013/2014–2024/2025 (excl. 2020/2021, 2021/2022) | COVID-19 pandemic seasons excluded: 2020/2021 because reported positivity ran at or near zero across all entities in a manner attributable to pandemic reprioritisation of surveillance (treated as unreliable rather than true low circulation), and 2021/2022 for disrupted reporting and divergent epidemic timing across the compared entities (peak-week separation far larger than in any analysed season). See Methods Section 2.2. 2019/2020 truncated after W5. |
| **Number of weeks per season** | 33 (W40–W20) | Northern Hemisphere convention; standard ECDC/WHO-Europe surveillance window. |
| ***Epidemic Timing*** | | |
| **Method** | Fixed criterium (i.method = 2; package default) | Identifies the epidemic period via the MAP curve slope. Chosen over the original, slope and second-derivative alternatives because it is the variant described by Vega et al. (2013) and implemented for ECDC and WHO-Europe reporting, and because it exposes a single interpretable tuning parameter that can be optimized against observed classification performance. |
| **Slope parameter** | 1.8 (Türkiye, WA); 2.3 (SWE) — i.param. NOT a default: the mem package default is 2.8 | The only empirically chosen parameter. Optimized independently per entity via MCC maximisation over the range 1.0–5.0 in steps of 0.1 (roc.analysis, i.param.values = seq(1, 5, 0.1)) under cross-validation. The optimized values are below the package default of 2.8 and below the 2.5–3.0 range described as typical in the package documentation; because the fixed criterium places the epidemic boundary where the MAP curve slope falls below this value, a lower value lengthens the identified epidemic period. |
| **Centering** | MEM automatic centring (i.centering = -1; package default) | Seasons are aligned on their epidemic periods, with the number of centring weeks selected automatically by MEM. |
| ***Epidemic Threshold*** | | |
| **Pre-epidemic values per season** | Automatic (i.n.max = -1; package default); effective value returned = 3 | Implements the published rule: Vega et al. (2013) specify taking the highest n values per season where n = 30 / (number of seasons). With 10 seasons this gives 3 values per season and 30 pooled pre-epidemic values. The resulting sample of 30 is not incidental — it is the conventional point at which a normal approximation becomes acceptable, which is also why the package flags the t-distribution option below 30 values. |
| **Threshold calculation** | Arithmetic mean + point CI (i.type.threshold = 5; package default). Threshold = upper one-sided limit only, i.e. mean + z·SD | Both parts of this choice are substantive. Arithmetic rather than geometric is required by the data: 47% of Türkiye's W40-W47 observations across the analysed seasons are exactly zero, and a log-scale estimator is undefined in the presence of zeros. A point interval built from the standard deviation, rather than a mean interval built from the standard error, is required because the threshold must discriminate an individual week against the spread of baseline weeks, not locate the mean of the baseline; an interval based on the standard error would narrow as seasons accumulate and the threshold would drift downward over time. One-sided tails follow from the threshold being directional. This is the estimator specified in Vega et al. (2013), whose upper limit is defined there as the epidemic threshold. |
| **Confidence interval tails** | One-sided, upper (i.tails.threshold = 1; package default) | One-sided CI provides a single upper threshold; consistent with the objective of detecting when activity exceeds baseline. |
| **Confidence level** | 95% (i.level.threshold = 0.95; package default) | Standard epidemiological significance level. |
| ***Intensity Thresholds*** | | |
| **Number of values per season** | Automatic (i.n.max = -1; package default); effective value returned = 3 | MEM automatically selects; 3 peak values/season × 10 seasons = 30 epidemic values pooled. |
| **Threshold calculation** | Geometric mean + point CI (i.type.intensity = 6; package default). Thresholds = upper one-sided limits computed on the log scale as log-mean + z·SD(log) and back-transformed | Geometric mean is appropriate and available here because the values entering this calculation are seasonal peak positivity values, which are strictly positive and characteristically right-skewed; the zero-value problem that governs the epidemic threshold estimator does not arise. Matches the estimator specified in Vega et al. (2013). |
| **Confidence interval tails** | One-sided, upper (i.tails.intensity = 1; package default) | One-sided CI provides upper thresholds for each intensity level. |
| **Intensity levels (CI %)** | Medium 40%, High 90%, Very high 97.5% (i.level.intensity = c(0.4, 0.9, 0.975); package default) | These are the levels harmonised across the WHO European Region and used in contemporary reporting and the WHO PISA framework, adopted so that intensity classification is comparable with current European practice. They deliberately differ from the 50%, 90% and 95% levels used in the original Vega et al. (2013) analysis. |
| ***Average Curve and Other Estimators*** | | |
| **Average curve CI method** | Geometric mean + mean CI, two-sided 95% (i.type.curve = 2, i.level.curve = 0.95; package defaults) | Geometric mean accounts for skewness in weekly rates. A two-sided interval is used here, unlike the thresholds, because the average curve describes a central estimate rather than a detection boundary and therefore requires both bounds. |
| **Other estimators CI method** | Median + Hettmansperger–Nyblom CI, two-sided 95% (i.type.other = 3, i.level.other = 0.95; package defaults) | Epidemic start, duration and percentage are estimated from only 10 seasons and are vulnerable to outlier seasons — 2014/2015 peaked in W11 against a typical W1 — so a non-parametric location estimator with a distribution-free interval is preferable to a mean. |
| ***Validation and Optimization*** | | |
| **Validation scheme** | Cross-validation (i.goodness.method = "cross"; package default) | Each season is tested against thresholds derived from all other seasons, preceding and subsequent. Sequential validation was rejected because, with 10 seasons and a 6-season minimum for model fitting, the earliest seasons could not have been evaluated at all. Note that cross-validation measures the internal consistency of the threshold across the historical series and does not estimate prospective real-time detection performance. |
| **Optimization metric** | Matthews Correlation Coefficient (MCC) | MCC provides a balanced measure of classification quality suitable for unequal class sizes (epidemic vs. non-epidemic weeks). |
| **Optimization range** | Slope 1.0–5.0, step 0.1 (i.detection.values = seq(1, 5, 0.1); package default) | Covers the full recommended parameter space per MEM documentation. |
| **Bootstrap parameters (not operative)** | i.type.boot = "norm", i.iter.boot = 10000 (package defaults) | Retained at package default but not operative in this analysis: bootstrap intervals apply only when a median-and-bootstrap estimator is selected (type 4), which was not used for any quantity here. Confidence intervals were based on z values rather than t values (i.use.t = FALSE; package default); the package recommends the t option where fewer than 30 values are used, and exactly 30 pooled values were available here. This is noted in the Limitations. |

**Supplement Figure S1.**

**Figure S1.** Weekly influenza positivity rates from ILI-based sentinel surveillance, Türkiye, 2013/2014 to 2024/2025 (epidemiological week 40 to week 20). Each line represents one influenza season. The two COVID-19 pandemic seasons (2020/2021 and 2021/2022) were excluded from the MEM analysis but are shown here for completeness; the 2019/2020 season is truncated after week 5. Positivity rate = influenza-positive specimens / specimens tested x 100. Source: WHO FluNet.

**Supplement Note S1. Software and reproducibility**

All analyses were performed with the Moving Epidemic Method as implemented in the mem package for R (see Resources section below), operated through its graphical front end, the MEM Shiny web application memapp (see Resources section below). The complete model specification is given parameter by parameter, with the corresponding mem argument names, in Supplement Table S4, so that every reported value can be reproduced. Rather than distribute a bespoke analysis script, readers are directed to memapp, for the reasons set out below.

**Why the web application rather than a code listing**

An annotated R code listing was initially prepared for this supplement. Reproducing the analysis from such a listing would, however, require a reader to carry out several technical preparatory steps outside the method itself: arranging the weekly series into the one-column-per-season matrix MEM expects, accommodating week 53, coding missing weeks correctly (blank rather than zero), and invoking the model, goodness and optimization functions with the correct arguments. The memapp web application performs all of these steps through a point-and-click interface: it imports data from common formats (CSV, Excel, and others), applies the required transformations automatically, exposes every parameter used here as a labelled option, and produces the thresholds, goodness indicators, optimization output and figures directly. Directing readers to memapp therefore serves reproducibility at least as well as a script would, while lowering the barrier for readers new to the method who wish to apply it to their own surveillance data. This choice reflects the study's aim of promoting uptake of standardised threshold estimation, not only of documenting a single analysis.

**Accessing and running memapp**

memapp is free and open source. It can be installed from CRAN within R with install.packages("memapp") and launched with memapp::runmemapp(launch.browser = TRUE); the development version is available from the project repository. A public instance is also hosted by the developers and can be used directly in a web browser without any local installation. The repository landing page provides, in dedicated sections, the installation instructions, usage notes, additional package notes, the technical manual (stable and development versions), useful links (including the CRAN page and the hosted server), and the reference list for the method.

**Resources**

Project repository (installation, usage, notes, technical manual, useful links, references):

mem R package (CRAN): <https://CRAN.R-project.org/package=mem>

MEM Technical Manual [7]: <https://github.com/lozalojo/memapp>

Source surveillance data: WHO FluNet, <https://www.who.int/tools/flunet>

The weekly positivity series analysed here are provided in Supplement Tables S1-S3; the full model specification is in Supplement Table S4.

**Reproducing the reported analysis in memapp**

To reproduce the results, load the weekly positivity series for the entity of interest (Supplement Tables S1-S3), set the options exactly as listed in Supplement Table S4 - fixed criterium timing with the entity-specific slope parameter (1.8 for Türkiye and Western Asia, 2.3 for South West Europe), 3 values per season (30 pooled values), one-sided thresholds, arithmetic-mean epidemic threshold, geometric-mean intensity thresholds at 40/90/97.5%, cross-validation, and MCC as the optimization metric over the 1-5 range - and read the epidemic and intensity thresholds, the goodness indicators and the average curve from the corresponding tabs. Because the slope parameter is optimized on the full-precision surveillance series, the optimization step reproduces 1.8 for Türkiye; note that the MCC surface is flat in the neighbourhood of the optimum, so the derived thresholds are insensitive to small changes in the slope.
